# The Factors of Adolescent Depression in Jiangsu Province, China, from the Perspective of Social Ecosystem: A Case-Control Study

**DOI:** 10.1155/2023/2861629

**Published:** 2023-11-11

**Authors:** Hua Xu, Zhao-hong Chen, Jin-jie Ji, Juan She, Chun-ting Hou, Yan-hong Zhang

**Affiliations:** ^1^Department of Adult Psychiatry, The Affiliated Brain Hospital of Nanjing Medical University, Nanjing, China; ^2^Department of Early Intervention, The Affiliated Brain Hospital of Nanjing Medical University, Nanjing, China; ^3^Department of Nursing, The Affiliated Brain Hospital of Nanjing Medical University, Nanjing, China

## Abstract

**Background:**

The prevalence of adolescent depression is continuously increasing, impacting the cognitive, emotional, and behavioral development of adolescents. Therefore, it is crucial to comprehend depression from the perspective of the social ecosystem, necessitating further empirical evaluation.

**Methods:**

This study utilized a case-control study design. The cases consisted of adolescents with depression (aged 13–18 years) admitted to a psychiatric hospital in Nanjing, Jiangsu Province, China, from November 2021 to July 2022, meeting the diagnostic criteria for depression in the 11th Edition of the International Classification of Diseases. Control group students, with a matching gender ratio in the same region, were randomly recruited. Logistic regression analysis was employed to investigate the factors associated with depression, with gender and age as covariates.

**Results:**

The study comprised 200 participants, with 44 (22.0%) males and 156 (78.0%) females. Multiple logistic regression analysis revealed that increased adolescent depression was associated with being an only child (AOR = 2.680, 95% CI: 1.106–6.492), living in urban areas (AOR = 3.324, 95% CI: 1.077–10.267), experiencing school bullying (AOR = 9.087, 95% CI: 2.044–40.408), having severe family dysfunction (AOR = 6.491, 95% CI: 1.109–37.995), and possessing low core self-evaluation (AOR = 11.746, 95% CI: 3.305–41.746). Odds ratios for each factor were statistically significant.

**Conclusions:**

Our results improve the evidence for associations between adolescent depression and core self-evaluation, school bullying, family function, living in urban areas, and being an only child. These findings should be taken into consideration in the assessment, intervention, and related policies for adolescent depression.

## 1. Introduction

Depression is a prevalent mental health disorder among adolescents, with its prevalence steadily rising, particularly following the sudden outbreak and rapid spread of COVID-19 in China [1]. During the COVID-19 pandemic, Chinese adolescents have faced substantial challenges in maintaining their mental well-being, resulting in a higher incidence of depression, reaching 36.6%-43.7% [2, 3]. The complex socioeconomic and educational changes, coupled with shifts in family structures, further exacerbate the prevalence of depression and its detrimental impact on various aspects of life [4]. Depression significantly hampers the psychosocial functioning of adolescents, affecting their academic performance and social interactions, leading to diminished self-esteem and overall well-being [5]. Major depressive episodes during adolescence also disrupt cognitive, emotional, and behavioral development, increasing the risk of suicidal tendencies [6]. Given the escalating incidence and severity of adolescent depression, early identification of contributing factors and the implementation of preventive strategies for at-risk adolescents are paramount in addressing this global issue [7].

Research suggests that family-related factors are primary drivers of adolescent depression [8]. The family environment, including parent-child relationships and family breakdown or discord [9], along with adverse life events such as school bullying and negative parenting styles [10, 11], are linked to an increased risk of adolescent depression. Psychosocial elements, like mental resilience and social support, play a pivotal role in modulating various systems and mitigating depressive emotional problems in adolescents [12]. Sociodemographic characteristics are also associated with adolescent depression in China [13], with debates over the correlation between being an only child and adolescent depression. Sun et al. [14] found no such correlation, while gender was a significant determinant of adolescent depression. Another study indicated that girls from nononly-child families and children from only-child families with poor family function were more likely to exhibit depressive symptoms [15]. Social support from parents and peers acts as a protective factor throughout an individual's life [16]. Despite extensive international research on the factors influencing adolescent depression, variations based on cultural backgrounds and ethnicities have been observed [17]. Moreover, environmental disparities may affect the feasibility of measuring predictors. Many studies have not prioritized clinical diagnosis of depression as their primary outcome. Consequently, it is essential to explore risk predictors of adolescent depression from a global perspective, particularly for individuals residing in low- and middle-income countries [18].

As the COVID-19 pandemic continues to spread globally, China's focus on epidemic prevention has shifted from emergency control to regular prevention and management. However, the daily lives of people, particularly adolescents, have been significantly disrupted, affecting their ability to engage in social activities and impacting their mental well-being, especially those already experiencing depression. This has exacerbated their depression and anxiety [19]. While several studies have investigated the mental health of healthy adolescents during the ongoing COVID-19 pandemic, the connection between these factors and adolescents with depression remains unclear. Therefore, it is imperative to investigate factors related to adolescent depression in the context of a major public health emergency, such as the novel coronavirus pneumonia epidemic, to provide a theoretical foundation for addressing the mental health needs of adolescents with depression during such crises.

In this study, we employed the social ecosystem model as a theoretical framework [20] and adopted a model-thinking approach to explore predictors of adolescent depression. Within this framework, we assessed predictors affecting adolescent depression from three perspectives: microsystem, mesosystem, and macrosystem, providing valuable insights for clinical treatment and intervention. Consequently, we formulated the following hypotheses: (1) in the microsystem, high core self-evaluation and psychological resilience in adolescents serve as protective factors against depression, while being an only child is associated with adolescent depression. (2) In the mesosystem, good family functioning and healthy relationships with parents and peers act as protective factors, while living in urban areas and experiencing school bullying are independent predictors. (3) In the macrosystem, gaining social support is a protective factor for adolescent depression.

## 2. Methods

### 2.1. Participants

A total of 100 adolescent patients diagnosed with depression and admitted to Nanjing Brain Hospital between November 2021 and July 2022 were categorized into the case group. All patients received mood stabilizers or antidepressants, such as fluoxetine, venlafaxine, and lithium carbonate. The inclusion criteria for the case group were as follows: (1) aged 13–18 years, (2) a Hamilton depression rating scale (HAMD) score ≥ 8, (3) a diagnosis of depression meeting the criteria of the 11th Edition of the International Classification of Diseases [21], and (4) no comorbid mental disorders (e.g., attention-deficit/hyperactivity disorder, autism, and eating disorders). Individuals were excluded from the case group if they met the following criteria: (1) underwent modified electroconvulsive therapy or systemic psychotherapy during the study period, (2) had concurrent physical brain disorders or organic brain syndrome, (3) had a history of severe alcohol or substance misuse, or (4) had a history of manic or hypomanic episodes.

Recruitment information was also disseminated within the hospital, and students from the same region with a matching gender ratio to the case group were randomly recruited as the control group. The sample was recruited using a consecutive sampling method employing a successive simple method. A total of 100 adolescents were included. The inclusion criteria for the control group were as follows: (1) aged 13–18 years, (2) absence of neurological or mental diseases, and (3) a HAMD score < 8. The exclusion criteria for the control group included (1) severe physical illness, alcohol or drug abuse, and behavioral disorders or (2) a family history of mental illness spanning more than three generations.

### 2.2. Sample Size Calculation

We conducted a case-control study to determine the sample size [22]. Based on previous reports [15], the prevalence of depression in Chinese adolescents is 35%. For our calculations, we utilized the probability of exposure in sampled control individuals (P0) = 0.35, the odds ratio (OR) representing the strength of the association between exposure factors and depression = 3.5, the correlation of exposure among individuals (Phi) = 0.2, Alpha = 0.05, and *β* = 0.10. These values were entered into PASS 21.0 software to determine a required sample size of 69 participants. To account for a potential 20% loss to follow-up, each group required a minimum sample size of 83 participants. Ultimately, we enrolled 100 adolescents in each group.

### 2.3. Ethical Considerations

This study received approval from the hospital's medical ethics committee. Participants with depression provided written informed consent, and they were informed that they could withdraw from the study at any time without affecting their children's clinical care. Informed consent was also obtained from healthy control participants and their guardians, and all personal information of the participants was kept confidential.

### 2.4. Data Collection and Quality Control

Data collection for the case group was carried out within the ward, with study group members administering the questionnaires in person. Participants received clear instructions and precautions before the assessment, emphasizing the absence of right or wrong answers in the questionnaire. They were encouraged to answer truthfully based on their own experiences and not to consult with others. Researchers were present to address any participant questions, and honesty in responses was encouraged.

Data collection for the healthy control group was conducted at school or in the hospital. Researchers provided a letter to the parents of recruited adolescents explaining the study's purpose and obtained informed consent from both the adolescents and their parents. Before data collection, the adolescents were assessed for mental health by psychiatrists and then completed self-reported questionnaires. Teachers and parents were asked to leave during this process. Researchers collected the questionnaires, and participating students received compensation in the form of a gift.

Experienced and high-quality investigators conducted quality control of the collected questionnaires. We used EpiData 3.1 to construct the database, employing a double data entry approach to verify data consistency.

### 2.5. Measures

All evaluation questionnaires consisted of self-rating scales assessing various sociodemographic parameters and clinical scales, including the adolescent core self-evaluation, family function, child and adolescent social support, and resilience for adolescents.

A self-designed sociodemographic information questionnaire included items on age, gender, only child, place of residence, school bullying, relationships with parents, and relationships with classmates.

The Chinese version of the core self-evaluation scale (CSES), revised by Du [23], was used to measure the core self-evaluation of adolescents in both groups. The scale comprises 10 items, each rated on a 5-point scale from “strongly disagree” to “strongly agree,” with scores ranging from 1 to 5. The total adolescent CSES scale score ranges from 10 to 50 points, with higher scores indicating higher core self-evaluation. The norm for core self-evaluation in Chinese adolescents was set at 31 points. In this study, a score of ≤31 points indicated low core self-evaluation, while a score of >31 points indicated high core self-evaluation.

The family APGAR index (FAI), developed by Dr. Smilkstein [24] and adopted in China, was used to assess participants' family function. The FAI consists of five components: family well-being, collaboration, development, emotional state, and closeness. A 3-point Likert scale was used for assessment, with “frequently” assigned 2 points, “occasionally” assigned 1 point, and “infrequently” assigned 0 points. The cumulative score was obtained by summing the scores of the five components and providing an indication of the participant's satisfaction with their family function. A total score of 7–10 points indicated good family function, a score of 4–6 points indicated moderate family dysfunction, and a score of 0–3 points indicated severe family dysfunction.

The child and adolescent social support scale, developed by Ye and Dai [25], was employed to measure the perceived support of the participants. This scale consists of three dimensions: subjective support, objective support, and support utilization, comprising a total of 17 items. It employs a 5-point scoring system, with “agree” representing 5 points, “somewhat agree” representing 4 points, “not sure” representing 3 points, “somewhat disagree” representing 2 points, and “not agree” representing 1 point. The total score on the scale ranges from 17 to 85 points, with higher scores indicating a higher level of social support and its dimensions. The social support norm for Chinese adolescents was set at 61 points. In this study, a score of ≤61 points was considered low social support, and a score of >61 points was considered high social support.

The resilience scale for adolescents, designed by Hu and Gan [26], was used to measure the psychological resilience level of the participants. The scale includes 27 items that focus on five factors: goal focus, emotional control, positive cognition, family support, and interpersonal assistance. Each factor comprises 4–5 items. The scale employs a 5-point scoring system, with the overall score ranging from 27 to 135 points. A higher score on the scale indicates a higher level of psychological resilience. The mean score for psychological resilience among Chinese adolescents was 90.12 points. In this study, a score of ≤90 points was classified as low psychological resilience, while a score exceeding 90 points was categorized as high psychological resilience.

### 2.6. Data Analysis

Data analysis was conducted using SPSS 27.0. The normality of measurement data was assessed using the Kolmogorov-Smirnov test. Measurement data that followed a normal distribution were presented as mean ± standard deviation ($\bar{x}\pm s$), and differences between groups were compared using the *t*-test. Enumeration data were described as the number of cases (%), and between-group differences were compared using the *χ*^2^ test. All variables were categorized, and factors that were significant in univariate analysis were evaluated in the binary logistic regression model for both univariate and multivariate analyses. Collinearity between independent variables was tested before conducting logistic regression analysis. Linear regression was used to determine tolerance and VIF to establish the absence of multicollinearity between variables (Tolerance > 0.1 and VIF < 10 indicate no multicollinearity). Odds ratios (ORs) and 95% confidence intervals (CIs) were calculated using adjusted logistic regression models. A significance level of *P* < 0.05 was considered statistically significant.

## 3. Results

### 3.1. Descriptive Analysis of Demographic Data of the Two Groups

A total of 200 participants were enrolled in the study. The HAMD score for 100 adolescents in the case group was 32.43 ± 12.52, with a maximum of 58 and a minimum of 15. The case and control groups had 22 males (22.0%) and 78 females (78.0%) individually. The average age of participants in both groups was 15.25 ± 1.48, 15.00 ± 1.33, *t* = −1.258, and *P* = 0.210 years. Among the research subjects, 46.5% were only-child children, and the participants' places of residence were 53.5% in urban areas, 24.0% in towns, and 22.5% in rural areas. In the case group, 35.0% of participants experienced school bullying. A chi-square test revealed that only-child status, place of residence, relationships with parents, relationships with classmates, and school bullying were associated with adolescent depression, as shown in Table 1.

### 3.2. Comparison of Core Self-Evaluation, Social Support, Family Function, and Psychological Resilience between the Two Groups

An independent sample *t*-test demonstrated that the case group and the control group differed significantly in adolescent core self-evaluation (25.83 ± 7.50, 38.99 ± 6.782, *t* = 13.017, *P* ≤ 0.001), adolescent social support (54.87 ± 16.55, 72.37 ± 11.68, *t* = 8.637, *P* ≤ 0.001), psychological resilience (79.42 ± 18.56, 101.46 ± 18.45, *t* = 8.423, *P* ≤ 0.001), and family function (5.03 ± 2.82, 7.59 ± 2.35, *t* = 6.976, *P* ≤ 0.001). A chi-square test revealed statistically significant differences between the two groups in adolescent core self-evaluation, family function, perceived social support, and adolescent psychological resilience, as shown in Table 2.

### 3.3. Univariate and Multivariate Analyses of Adolescent Depression

A diagnosis of depression was set as the dependent variable (no = 0; yes = 1), and variables that were significant in univariate analysis were set as independent variables, which were included in multivariable logistic regression models for adjustment (Table 3). The results indicated no collinearity among the independent variables, with each variable exhibiting a tolerance greater than 0.1 and a VIF less than 10. After statistical testing, the logistic regression model was found to be significant (model *χ*^2^ = 138.746, *P* < 0.001).

Table 3 revealed the association between adolescent depression and variables such as only-child status, place of residence, school bullying, relationships with parents and classmates, family function, adolescent core self-evaluation, social support, and psychological resilience, along with their adjusted odds ratios (AOR) and crude odds ratios (COR). Logistic regression findings unveiled that, in comparison to non-only-child children, adolescents who were only-child children exhibited higher odds of experiencing depression (AOR = 2.680, 95% CI: 1.106–6.492, Wald = 4.768, *P* = 0.029). Concerning place of residence, adolescents residing in urban areas were found to have approximately threefold higher odds of depression compared to those in rural areas (AOR = 3.324, 95% CI: 1.077–10.267, Wald = 4.360, *P* = 0.037). In terms of family function, adolescents facing severe family dysfunction displayed a higher prevalence of depression (AOR = 6.491, 95% CI: 1.109–37.995, Wald = 4.304, *P* = 0.038). Those who experienced school bullying exhibited increased odds of depression compared to those who had not (AOR = 9.087, 95% CI: 2.044–40.408, Wald = 8.403, *P* = 0.004). Furthermore, a strong association was noted between low core self-evaluation and adolescent depression (AOR = 11.746, 95% CI: 3.305–41.746, Wald = 14.496, *P* < 0.001).

## 4. Discussion

This study examined factors associated with adolescent depression across three systemic levels: microsystem, mesosystem, and macrosystem. We identified that the factors most closely related to adolescent depression were only child, adolescent core self-evaluation, place of residence, school bullying, and family function. However, it remains to be determined whether psychological resilience, social support, relationships with classmates, and relationships with parents are associated with adolescent depression.

Our findings indicated that only-child children were 2.680 times more likely to develop depressive symptoms compared to non-only-child children. This result was consistent with our hypothesis but contradicted a previous study [15]. The incidence of depression among adolescents residing in urban areas was found to be 3.324 times higher than those living in rural areas. This finding aligns with the results of a recent study [27]. One possible explanation could be that the study participants were recruited from Jiangsu, an economically developed province in China, where adolescents may be more susceptible to depressive symptoms due to the fast-paced nature of modern cities and the intense academic competition and pressure to succeed academically. The implementation of a series of prevention and control measures for COVID-19, such as lockdowns in urban areas, traffic control, school-home two-point measures, and more strict and prolonged restrictions in urban areas, may have contributed to longer-term loneliness, loss of close peer relationships, and lack of outdoor physical activities [28].

Core self-evaluation refers to an individual's assessment of their own abilities and self-worth [29]. During adolescence, core self-evaluation is influenced by situational and external factors [30]. For example, when adolescents encounter negative assessments such as neglect, exclusion, or harsh criticism, they may internalize these unfavorable judgments into their self-concept, leading to diminished self-esteem and self-assessment. We found that adolescents with low self-evaluation were 11.746 times more likely to develop depression than those with high self-evaluation. Core self-evaluation plays a significant role in predicting mental health and acts as a mediator between stressful life events and depression, as well as between suicidal ideation and depression [31, 32]. Specifically, individuals with low core self-evaluation may struggle to cope with the challenges they face, increasing their vulnerability to depression and potentially inducing suicidal thoughts or behaviors in adolescents with depression. The study suggests that psychotherapy can correct patients' negative cognitions and improve their symptoms and prognosis. For instance, cognitive therapy can help individuals correct irrational beliefs and change maladaptive schemas, leading to a more positive self-view and reduced sensitivity to nonhostile behaviors of others as potential ostracism [33]. Ultimately, this intervention may reduce the risk of depression.

Environmental influences play a significant role in shaping the growth, character, and abilities of adolescents. Among these influences, both the school environment and family environment emerge as pivotal systems that affect the maturation and development of adolescents. Our results have demonstrated an association between school bullying and adolescent depression. School bullying stands as a noteworthy challenge faced by numerous adolescents within the school environment, and it is linked to issues of mental well-being and inadequate socioemotional adaptation within the educational setting [34]. The concealment and persistence of being bullied could induce depression and persistent negative emotions in the affected adolescents [34]. In situations where protective factors are lacking or limited, long-term exposure of an adolescent to such chronic stress may impair the person's emotional and cognitive development, rendering them more susceptible to developing depressive symptoms [35]. Therefore, educators should formulate targeted measures to prevent school bullying or provide corresponding intervention based on the specific situation of local/school bullying to enhance the psychological well-being of affected adolescents and create a harmonious and friendly school environment. Within the framework of adolescent mental health education, it is imperative to provide guidance to teenagers regarding the cultivation and sustenance of constructive friendships with peers.

As predicted, there was a significant association between family function and adolescent depression, with severe family dysfunction predicting a higher prevalence of depression. According to previous studies, adolescents with poor family function are more likely to experience psychological disorders such as depression, fear, and anxiety [10, 36]. Good family function positively predicts positive emotions in adolescents [37]. Families with high levels of dysfunction typically exhibit lower ability to communicate with each other and solve problems [38]. Consequently, in families marked by dysfunction, adolescents face challenges in effectively expressing their emotions and thoughts to their parents. This inability can subsequently impede their access to sufficient familial support during times of necessity, potentially resulting in psychological issues such as anxiety and depression. Therefore, to prevent adolescent depression, parents should foster a positive family atmosphere and increase emotional communication with their children [39]. At the same time, the more care the family gives to the young, the easier it is for the children to obtain a sense of security and belonging, to receive effective understanding and support from the family when they encounter difficulties, and to avoid the development and spread of negative emotions [40]. However, this requires parents to make corresponding changes in parent-child communication, emotional connection, and problem coping [41]. Therefore, family therapy involving parents is essential in the intervention for adolescent depression.

In Table 3, the univariate logistic regression results showed that social support, psychological resilience, and depression are correlated. Regrettably, our multiple logistic regression analysis did not reveal a correlation between social support and psychological resilience with adolescent depression. However, previous studies [16, 42] have shown that social support, psychological resilience, and depression are correlated. The reason for this difference is that, in addition to the different subjects, this study only measures the level of social support and psychological resilience of adolescents from a single perspective and simple scale tools. Therefore, different multiperspective and multicenter research methods are needed to further evaluate the association between social support, psychological resilience, and depression in adolescents with depression.

## 5. Limitations

The present study has several limitations. First, this study relied on self-report data and could thus be influenced by reporting and memory biases. Second, this study had a case-control design, and the temporal relationship between exposure and outcome was uncertain, needing further validation through prospective cohort studies. Furthermore, this sample is indicative of a specific segment from Jiangsu Province, China, and its generalizability to diverse regions is limited. As a result, our future work should build on this research with an expanded sample size and conduct long-term follow-ups to examine the effect on the development of anxiety and depression.

## 6. Conclusions

Following adjustments for gender and age, our investigation revealed an association between adolescent depression and variables, including school bullying, diminished self-assessment, pronounced family dysfunction, and residing in an urban locale. These findings provide insight into the social-ecological mechanisms underlying adolescent depression. Further clinical studies should focus on screening and preventing the affected groups, providing early intervention and treatment by targeting predictors for depression.

## Figures and Tables

**Table 1:** Univariate analysis of demographic data, school bullying, relationships with parents, and relationships with classmates of the two groups (*n* = 200) (*t*/*χ*^2^, *n* (%)).

Variable	Case (*n* = 100)	Control (*n* = 100)	*t*/*χ*^2^	*P*
Age	15.25 ± 1.48	15.00 ± 1.33	-1.258	0.210
Gender				
Male	22 (22.0%)	22 (22.0%)	0.000	1.000
Female	78 (78.0%)	78 (78.0%)		
Only child				
No	41 (41.0%)	66 (66.0%)	12.562	≤0.001
Yes	59 (59.0%)	34 (34.0%)		
Place of residence				
Urban areas	65 (65.0%)	42 (42.0%)	10.633	0.005
Town	18 (18.0%)	30 (30.0%)		
Rural areas	17 (17.0%)	28 (28.0%)		
School bullying				
No	65 (65.0%)	96 (96.0%)	30.610	≤0.001
Yes	35 (35.0%)	4 (4.0%)		
Relationships with parents				
Bad	12 (12.0%)	3 (3.0%)	38.184	≤0.001
Average	38 (38.0%)	7 (7.0%)		
Good	50 (50.0%)	90 (90.0%)		
Relationships with classmates				
Bad	10 (10.0%)	1 (1.0%)	36.777	≤0.001
Average	43 (43.0%)	12 (12.0%)		
Good	47 (47.0%)	87 (87.0%)		

**Table 2:** Comparison of core self-evaluation, social support, family function, and resilience scale between the two groups (*n* = 200) ($\bar{x}\pm s$,*t*/*χ*^2^, *n* (%)).

Variable	Case (*n* = 100)	Control (*n* = 100)	*t*/*χ*^2^	*P*
Core self-evaluation	25.83 ± 7.50	38.99 ± 6.78	13.017	≤0.001
Low-score group	77 (77.0%)	13 (13.0%)	82.747	≤0.001
High-score group	23 (23.0%)	87 (87.0%)		
Adolescent social support	54.87 ± 16.55	72.37 ± 11.68	8.637	≤0.001
Low-score group	63 (63.0%)	14 (14.0%)	50.702	≤0.001
High-score group	37 (37.0%)	86 (86.0%)		
Psychological resilience	79.42 ± 18.56	101.46 ± 18.45	8.423	≤0.001
Low-score group	80 (80.0%)	18 (18.0%)	76.911	≤0.001
High-score group	20 (20.0%)	82 (82.0%)		
Family function	5.03 ± 2.82	7.59 ± 2.35	6.976	≤0.001
Severe family dysfunction	28 (28.0%)	4 (4.0%)	37.093	≤0.001
Moderate family dysfunction	44 (44.0%)	29 (29.0%)		
Good family function	28 (28.0%)	67 (67.0%)		

**Table 3:** Univariate and multivariate analyses of adolescent depression.

Variable	Univariate analysis			Multivariate analysis			
	COR	95% (CI)	*P*	Wald	AOR	95% (CI)	*P*
Constant				24.861	0.045		<0.001
Only child							
No^★^							
Yes	2.793	1.573-4.962	<0.001	4.768	2.680	1.106-6.492	0.029
Place of residence							
Place: rural areas^★^							
Place: urban areas	2.549	1.2448-5.220	0.011	4.360	3.324	1.077-10.267	0.037
Place: town	0.988	0.427-2.288	0.978	0.114	1.247	0.346-4.493	0.736
School bullying							
No^★^							
Yes	12.923	4.383-38.104	<0.001	8.403	9.087	2.044-40.408	0.004
Relationships with parents							
Good^★^							
Average	9.771	4.064-23.491	<0.001	2.499	3.147	0.760-13.036	0.114
Bad	7.200	1.940-26.725	0.003	0.422	0.470	0.048-4.593	0.516
Relationships with classmates							
Good^★^							
Average	6.633	3.191-13.787	<0.001	1.858	2.272	0.698-7.398	0.173
Bad	18.511	2.299-149.063	0.006	0.831	3.290	0.254-42.568	0.362
Adolescent core self-evaluation							
High-score group^★^							
Low-score group	22.404	10.625-47.242	<0.001	14.496	11.746	3.305-41.746	<0.001
Family function							
Good family function^★^							
Moderate family dysfunction	3.631	1.907-6.911	<0.001	0.194	0.764	0.231-2.530	0.659
Severe family dysfunction	16.750	5.375-52.200	<0.001	4.304	6.491	1.109-37.995	0.038
Adolescent social support							
High-score group^★^							
Low-score group	10.459	5.217-20.970	<0.001	0.000	0.990	0.273-3.584	0.988
Psychological resilience							
High-score group^★^							
Low-score group	18.222	8.983-36.966	<0.001	0.046	1.162	0.297-4.551	0.830

★Reference.

## Data Availability

The datasets generated during and/or analysed during the current study are available from the corresponding author on reasonable request.
